# Annotations

**Published:** 1888-03-31

**Authors:** 


### Annotations.

**Irish Distressed Ladies.**

It will not surprise people who take interest in the general movements of the times that there are many ladies, both young and old, in Ireland who are reduced to poverty and distress. With the ladies must be included a large number of children, whose parents, once in prosperity, are not now able to give them education. In bringing before the notice of the public at the Mansion House the Fund which has been formed for the relief of our fellow-citizens across the Irish Sea, Lord Charles Beresford very wisely said that the audience should look at the circumstances as they were, and not regard the political causes which had brought about the distress. Therein he was surely in the right. Whatever view may be taken of Irish politics at the present crisis, no one can impute blame in the slightest degree to those ladies and children, many of whom through the diminution in the value of land, have been reduced from affluence to poverty. It was stated that since the formation of the Association 125 ladies over 70 years of age, or incapacitated in some other way, had been assisted, and there were still 105 on the books. Of those who were able to work 700 had been furnished with materials, such as needlework and sewing-machines, whilst 750 had been supplied with clothing. It ought not to be necessary to do more than mention such an association as this to ensure prompt and abundant support. There are few people more helpless, as a class, than educated women, trained to no occupation, who are suddenly reduced to want. There is no blame attributable to them for that. In these times of universal competition and struggle, many, even among the trained competitors, find it no easy task to keep the wolf from the door. What, then, are the chances of those who have no definite occupation, and no habits of self-reliance to fall back upon? There is abundance of superfluous wealth among the middle and upper classes, and these may be trusted to help at the present crisis those who have been nurtured as delicately and cared for as tenderly as themselves. For young, and even for middle-aged men there is always the army or a new country, and a hope, however small, of restored fortunes. But for women and children of the class under consideration there is practically no resource but the sympathy and aid of friends. The sum of £20,000 is asked for—not very much when the nature and extent of the distress are considered. We sincerely hope it may be provided within the next few weeks.

**Vegetarianism.**

In Dr. B. W. Richardson the Vegetarian Society has found an advocate who is able to do the fullest justice to its case. His knowledge, skill, and enthusiasm are all potent factors towards the advancement of any cause he may take in hand. The society has done well to formulate, to preach, and to practise its principles. Nothing is more deadening to men than routine, whether it be routine in food or in physic, in politics or in religion. A man may be as tightly locked up and imprisoned within a set of conventional ideas as within the most solid stone Bastille. Sometimes the very best thing possible for him is to be thrown violently and completely out of his "trivial round, his common task," and put upon his mettle and his manhood under an entirely new set of circumstances. We do not ourselves believe that the best diet for all men and women is a diet consisting exclusively of vegetables. Nor do we think, as some do, that many diseases result from flesh-eating which would be unknown under a vegetarian system. But it seems certain that many persons can live and be well on vegetables alone, that some are positively better in health under a vegetarian regimen, and that almost all would profit by a larger admixture and a greater variety of vegetable foods in their daily diet. Our protest is against the divinity of routine, the infallibility of conventionality, the loss and injury which grow from the tough and scirrhous roots of prejudice. A country physician of our acquaintance went to a vegetarian lecture three or four months ago. He went to scoff, but returned home to consider. He tried the system, beginning gradually, with perhaps a vegetarian dinner once or twice a-week. He and his wife are now almost entirely vegetarian in their habits. They are not "pledged" to the new rule exclusively, like "total abstainers"; but they practise it generally, and find in it gastronomic satisfaction, efficient health, and financial economy. We do not advise pure vegetarianism, because we object to fads of all kinds, and especially to those which involve a diminution of rational and moral liberty. But we do advise the intelligent consideration of Dr. Richardson's views on the subject, and such measures of practical experimentation as may lead to an enlarged, improved, and more economic dietary for all classes.

**"Sweating."**

The Committee appointed by the House of Lords to investigate the sweating system is in active operation. According to evidence given at last week's meeting a sweater can earn from 6*s*. to 8*s* a-day, working from eight in the morning to ten or twelve at night. Girls of sixteen or seventeen can earn 4*s*. a-day as button-hole makers, working, it may be presumed, the same length of time. Men earn at tailoring about 21*s*. a-week, but have to pay 5*s*. a-week for rent. The cases reported by Mr. Burnett of persons only earning 5*s*. or 6*s*. per week are said to be isolated and exceptional. Mr. Charles Booth is making an inquiry into the conditions of life and the occupations of the people at the East-end, which he states will occupy him six months. At present the flow of population is to London, but the flow of trade to the provinces. Mr. Booth had heard of a woman making four coats a-day, except the button-holes, for sevenpence a coat. The real facts, in their just proportions, it may be hoped and expected, will gradually be brought to light. Until that is done large measures of amelioration cannot be attempted. Even when the worst is known it is difficult to see what effectual steps can be taken except in the direction of diminishing the inflow of population to London. People of the poorest class, who think they have drunk the very dregs of life elsewhere, and that London may probably be better whilst it cannot possibly be worse than their present abode, rush to the metropolis as the last resource of despair. There is nothing at all surprising in this when it is remembered how hard and joyless is the life of a labourer in the country, even when work is plentiful; and how utterly hopeless it is when the one calling to which he has been bred ceases to furnish him with the means of providing daily bread for himself and his family. It ought to be possible to make poor men better acquainted with the actual conditions and prospects of their own class in the capital, and so to prevent a large number from adventuring themselves on such a stormy and desolate sea. Local authorities ought to recognise that we have a large movable population—a population always either on the wing, or ready to take wing on the slightest inducement. There should be some method of guiding their flight in the least harmful direction. Apart from the actual loss and injury to the body politic which result from the half-employment of vast masses of able-bodied people, our pride and our pity are alike hurt by the terrible spectacles of hopeless proverty and maddening recklessness which greet us on every hand. Many people are angrily ashamed of themselves and their fellow-countrymen, that, with a wealth compared with which that of all other ancient and modern kingdoms was as nothing, and with a race whose energy and practical capacity have made them masters and rulers of half the globe, we are not able to deliver ourselves from the grief and scandal of a starving and despairing population.

